# Guideline on the management of occupational and non-occupational exposure to the human immunodeficiency virus and recommendations for post-exposure prophylaxis: 2015 Update

**DOI:** 10.4102/sajhivmed.v16i1.399

**Published:** 2015-11-10

**Authors:** Michelle Moorhouse, Linda G. Bekker, Vivian Black, Francesca Conradie, Beth Harley, Pauline Howell, Gary Maartens, Tari Papavarnavas, Kevin Rebe, Gillian Sorour, Francois Venter, Carole L. Wallis

**Affiliations:** 1 Wits Reproductive Health and HIV Institute, Johannesburg, South Africa; 2 The Desmond Tutu HIV Centre, University of Cape Town, South Africa; 3 Right to Care and Clinical HIV Research Unit, University of Witwatersrand, South Africa; 4 City Health, City of Cape Town, South Africa; 5 Wits Health Consortium, University of Witwatersrand, South Africa; 6 Department of Medicine, University of Cape Town, South Africa; 7 Helen Joseph Hospital, Right to Care, South Africa; 8 Anova Health Institute, Johannesburg, South Africa; 9 Gauteng Department of Health, Gauteng, South Africa; 10 President's Emergency Plan for AIDS Relief, Wits Reproductive Health & HIV Institute, South Africa; 11 BARC, Johannesburg, South Africa; 12 Lancet Laboratories, Johannesburg, South Africa

## Abstract

This guideline is an update of the post-exposure prophylaxis (PEP) guideline published by the Southern African HIV Clinicians Society in 2008. It updates the recommendations on the use of antiretroviral medications to prevent individuals who have been exposed to a potential HIV source, via either occupational or non-occupational exposure, from becoming infected with HIV. No distinction is made between occupational or non-occupational exposure, and the guideline promotes the provision of PEP with three antiretroviral drugs if the exposure confers a significant transmission risk. The present guideline aligns with the principles of the World Health Organization PEP guidelines (2014), promoting simplification and adherence support to individuals receiving PEP.

## Key summary points

- Southern Africa differs from other regions, particularly in terms of very high HIV and hepatitis B virus (HBV) seroprevalence.
- Post-exposure prophylaxis (PEP) guidelines lack a substantive evidence base to guide advice. It is unlikely that this will change considerably, as randomised studies of different drug regimens for PEP are not feasible owing to the complexity of exposure, low event rate, and inability to ethically have a placebo group. Evolving basic science understanding, along with further studies on animals and prevention of mother-to-child transmission (PMTCT) findings, will continue to guide policy makers. In addition, data from pre-exposure prophylaxis (PrEP) studies will also provide valuable data relevant to PEP interventions.
- PEP guidelines prior to the Southern African HIV Clinicians Society's 2008 PEP guideline were not user friendly and rarely acknowledged the complex range of situations that occur with HIV.
- Selecting patients for appropriate PEP administration must be simplified. Algorithmic approaches for antiretroviral treatment (ART) regimens have simplified antiretroviral management at the treatment and management levels. The same approach is possible for PEP regimens in this region.
- The approach to occupational, sexual and other forms of HIV exposure (bites, assaults, trauma, injecting drug use, etc.) is similar.
- Cases of exposure are often not simple, do not lend themselves to simple categorisation, and require an individualised approach. However, concepts to guide the attending clinician are relatively simple and allow an effective intervention in most cases.

### Clinical approach

- Animal data, case control studies and PMTCT data suggest that PEP is highly effective if taken correctly for the full duration prescribed.
- Similarly, PrEP studies have indicated that, with high levels of adherence, PrEP is a highly effective intervention in the prevention of HIV transmission.
- The key outcome in HIV PEP is successful completion of one month of uninterrupted appropriate prophylaxis.
- Side-effect management is critical to completion, and is often under-managed. Zidovudine (AZT) and protease inhibitor (PI)-based regimens are associated with significant side-effects, and are therefore not preferred drugs in PEP regimens, except in special circumstances.
- The number of drugs used to treat PEP is often the focus of clinician attention. Whilst number of drugs and specific antiretroviral (ARV) prescribing are important, completing the full course, through active side-effect and anxiety management, remains the cornerstone of successful management.
- Side-effects owing to ART appear to be more common and severe in HIV-negative exposed people than in HIV-positive patients initiated on treatment, especially amongst healthcare workers (HCWs).
- There have been few documented failures of PEP. Many of these failures have been associated with poor adherence, suboptimal dosing or delayed ART.
- Anxiety management of the exposed individual must be actively addressed.

### Drug selection

- Where ART is felt to be justified, a three-drug regimen should be used. However, this must never be at the expense of adherence. Single- or dual drug regimens are known to be effective and can be used as an alternative where necessary (e.g. to increase adherence when a three-drug regimen is not well tolerated).
- The preferred nucleoside reverse transcriptase inhibitor (NRTI) backbone for PEP is tenofovir (TDF) with lamivudine (3TC) or emtricitabine (FTC), preferably as a fixed-dose combination (FDC).
- The preferred third drug is raltegravir (RAL).
- Owing to a lack of safety data regarding the use of RAL in pregnancy, atazanavir/ritonavir (ATV/r) is the preferred third drug in pregnancy.
- AZT-containing PEP regimens are associated with significant side-effects, whereas stavudine (d4T) is well tolerated for short-term administration; in patients where TDF cannot be used, d4T should be given preferentially to AZT.
- Nevirapine (NVP) should never be used for PEP owing to its potentially severe side-effects.
- Boosted PIs should be used in cases where ARV resistance is suspected, with NRTI choices based on medication to which the patient has not been exposed. Expert guidance should be sought in these situations.
- Hepatitis B prophylaxis, often not considered after HIV exposure, must form part of any assessment.
- Follow-up must be actively pursued. Advice on further HIV and hepatitis testing; when it is safe to commence unprotected sex; and subsequent primary prevention, are critical. Post-exposure HIV status should be assessed through serial enzyme-linked immunosorbent assay (ELISA) testing at 6 weeks and 3 months after exposure occurred. Polymerase chain reaction (PCR) testing does not currently have a role in PEP assessment.

### Public health issues

- Occupational exposure is usually avoidable. All cases should be investigated with a view to improving infection control.
- All health and allied institutions where exposure is an occupational risk should have clear, public and accessible PEP protocols.
- Hepatitis B vaccination programmes must be encouraged in all occupational health settings, as primary prophylaxis is very effective.

## Introduction

In 2008, the Southern African HIV Clinicians Society published guidelines on PEP, which were bold, specifically with regard to three key recommendations: the removal of the distinction between occupational versus non-occupational exposure; the use of triple prophylaxis; and treatment for all exposures.^1^ These points were in contrast to all other international guidelines at the time, and in fact it is only in the latest World Health Organization (WHO) PEP guideline that a similar approach to PEP has been promulgated.^2^

The present guideline updates the recommendations on the use of antiretroviral medications to prevent individuals who have been exposed to a potential HIV source, via either occupational or non-occupational exposure, from becoming infected with HIV. As in the 2008 guideline, no distinction is made between occupational or non-occupational exposure, and the guideline promotes the provision of PEP with three antiretroviral drugs if the exposure confers a significant transmission risk. There are strong recommendations with regard to the prevention of occupational exposure and the use of simplified approaches to PEP, with an emphasis on managing both the anxiety of the exposed individual, as well as a pro-active approach to side-effect management. See Table 1 for a summary of guideline recommendations.

**TABLE 1:** Summary of guidelines on post-exposure prophylaxis for HIV in adults, adolescents and children.

**Guideline**	**Recommendation**
Number of antiretroviral drugs	HIV PEP regimens should contain three drugs
Preferred PEP regimen for adults and adolescents	TDF + 3TC/FTC (preferably as fixed-dose combination) is recommended as preferred PEP backbone
	RAL is recommended as preferred third drug for PEP (except in pregnant women, where ATV/r is the recommended third drug)
	Alternative third drugs include ATV/r, LPV/r, DRV/r or EFV
Preferred PEP regimen for children ≤ 35 kg or unable to swallow tablets	AZT + 3TC is recommended as preferred backbone for HIV PEP in children ≤ 35 kg (substitute with d4T if AZT poorly tolerated)
	RAL is recommended as preferred third drug where available for HIV PEP in children. If RAL unavailable, then ATV/r is recommended
Prescribing frequency	A full one-month course of antiretroviral drugs should be provided for HIV PEP at initial assessment
	Starter packs should not be used
Frequency of follow-up	Exposed individual should be seen at 2 weeks, 6 weeks and 3 months after exposure occurred
Adherence support	Enhanced adherence counselling is recommended for all individuals initiating PEP

PEP, post exposure prophylaxis; TDF, tenofovir; 3TC, lamivudine; FTC, emtricitabine; RAL, raltegravir; ATV/r, atazanavir/ritonavir; LPV/r, lopinavir/ritonavir; DRV/r, darunavir + ritonavir; EFV, efavirenz; AZT, zidovudine.

Unfortunately, most of the data on which PEP guidelines are based are from different settings to the southern African region, and are largely derived from non-randomised control trial (RCT) data (except in the case of some of the PMTCT studies and more recently, the PrEP studies). Much of the data rely on retrospective register analysis, as well as extrapolation from animal data and individual clinical case studies. It is important to remember that these data from developed-world studies, where HIV epidemiology is significantly different and HIV prevalence considerably lower, may underestimate the risk of exposures in the southern African setting. However, due to ethical considerations and the numbers that would be needed to obtain RCT data, in the context of the relatively low rate of transmission associated with occupational exposure (if all parenteral exposures are considered together, the transmission risk is 0.3% per exposure),^3^ we are unlikely to obtain much additional or quality ’pure PEP’ data than what we currently have. In addition, PrEP studies consistently show that PrEP works when used properly, making it ethically questionable to conduct studies with PEP as a sole prevention intervention. In fact, there is a shift towards combination approaches to the prevention of HIV infection, of which PEP is only one component in a complex of prevention interventions.

Previous guidelines differentiated between occupational and non-occupational exposures. Given the very high background prevalence of HIV infection in the southern African region, HIV exposure risk outside the occupational setting is high, and the distinction between occupational and non-occupational exposure is less helpful for decision-makers. Further complicating the problem is the high rate of sexual assault in South Africa, and the large number of individuals with acute or primary HIV infection within the community. The generalised nature of the epidemic creates differences in risk group demographics that must be accommodated by local PEP guidelines. Finally, ‘non-traditional’ exposures, such as pre-mastication, tattoos, cuts from roadside barber's shears and other exposures listed below, often require clinician advice.

Whilst the actual management of exposure is the same whether the exposure was occupational or non-occupational, it is essential to document and manage occupational exposures appropriately, for possible subsequent compensation (including completion of the appropriate Compensation for *Occupational Injuries and Diseases Act* [COIDA] forms). This is also important in cases of sexual assault where legal and criminal proceedings may ensue.

**FIGURE 1:**
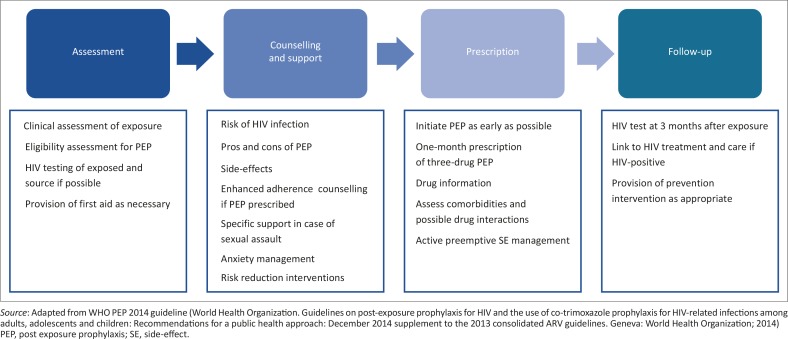
Care pathway for individuals exposed to HIV.

The present update to the PEP guidelines seeks to harmonise with the WHO PEP guidelines (2014) by alignment with WHO principles, and promoting simplification and adherence support to individuals receiving PEP (see Figure 1). The guidelines do not address PMTCT settings, PrEP or the comprehensive management of sexual assault. Local guidelines should be consulted as appropriate.

## Scale of the problem

About 3 million percutaneous exposures to bloodborne viruses occur globally amongst HCWs annually. A survey of more than 2400 USA HCWs showed that more than half had experienced a percutaneous injury in their career, and almost a quarter in the last year.^4^ A study in northern India demonstrated exceedingly high exposure rates, with 63% of participants reporting a percutaneous injury in the previous year, compared with the US data above.^5^ Mucocutaneous and percutaneous exposures over the previous week in the Indian study were reported at 11% and 30% respectively. These figures are not uncommon in lower income countries, with 55% of HCWs in Uganda and 57% of injection providers in Mongolia experiencing a percutaneous exposure in the last year.^6,7^

Data from the southern African region are limited and poor. The largest study from three West African countries documented that 45% of HCWs had sustained at least one accidental blood exposure, over 60% of which went unreported.^8^ In 2001, 69% of interns at Chris Hani Baragwanath Hospital in Gauteng, South Africa, had sustained at least one percutaneous injury, and 45% had sustained a mucocutaneous blood risk exposure.^9^ Again in this cohort, over 60% of exposures were not officially reported. At Tygerberg Hospital, Cape Town, 91% of junior doctors reported needlestick exposures in the prior year, three-quarters of these ‘after hours’ or during calls.^10^

Despite regulatory frameworks being in place in some countries, management oversight regarding occupational accidental blood exposure is largely lacking in southern African institutions, especially as far as the handling of sharps disposal and training in safe exposure practices are concerned.

In terms of non-occupational exposure, HIV transmission data for rape (a common experience for women, children and not a few men) are poor. There are almost no data on other forms of exposure; however, the continued high incidence and prevalence of HIV in southern Africa amongst the general population suggests that exposure is ongoing and high risk. Advice is frequently sought from clinicians regarding PEP following assault, traffic accidents and other trauma-related events where blood exposure occurs.

## Core principles of post-exposure prophylaxis

- Occupational exposure prevention requires strong management oversight in all settings.
- Non-occupational exposure requires an understanding of core transmission principles, combined with clinical common sense.
- In the southern African setting, all unknown source exposures should be assumed to be HIV-positive.
- Evidence regarding occupational and non-occupational risks of transmission for southern Africa is limited, and may underestimate transmission risk in our setting.
- Triple ARV regimens in treatment settings have been proven superior to mono or dual therapy regimens. However, in the setting of PMTCT and PrEP, mono and dual drug regimens have proven effective.
- It is recognised, however, that additional ARVs increase the potential side-effect and adherence burden. Risk of adverse effects and toxicities must be weighed against benefit in administering ARVs in the PEP setting. However, with increasingly well tolerated ARVs that are available, side-effects are becoming less of a problem. Nonetheless, side-effects must be treated rapidly, effectively and, where possible, avoided entirely. Ensure that the individual is aware of potential side-effects and has been advised how to deal with any that may arise. Patients should be advised that when in doubt they should rather see a healthcare provider as soon as possible and should only discontinue PEP under the guidance of a healthcare provider.
- PEP should be administered as soon as possible after exposure; efficacy after 72 hours is highly unlikely.
- To facilitate administering the first PEP dose as soon as possible after exposure, any ARV drug combination that is easily available can be used for the first dose, but the patient should not leave without a full month's supply (or prescription for a full month's supply) of the recommended regimen, namely TDF + FTC/3TC + RAL or TDF + FTC/3TC + ATV/r in pregnancy.
- Starter packs are not recommended owing to the high rate of default, as the exposed individual is often lost to care and does not return for the rest of the one-month supply.
- All PEP regimens must be administered for 28 days. Animal and case control studies suggest that administration for less than 2 weeks is associated with minimal efficacy; administration for more than 28 days confers no added benefit. Most ARVs are in packs of 30 tablets, and the full pack should be dispensed at the first visit.
- Regimens need to be selected using locally available ARVs.
- A comprehensive infrastructure of counselling and support for the exposed party is necessary to facilitate adherence to PEP regimens. Exposure is associated with substantial anxiety for most people; this must be dealt with actively. In many cases, anxiety is most significant for those who do not need PEP.
- Counselling must be available to deal with side-effects on an ongoing basis. AZT and PIs are commonly associated with side-effects.

### Balancing risks and benefits in post-exposure prophylaxis

People with HIV infection have a near-normal lifespan provided that ART is not started too late, so the risks of PEP need to be more carefully considered than in the past. On the other hand, newer antiretroviral drugs are considerably safer than most of the older agents. Most international guidelines on PEP, including those of the Southern African HIV Clinicians Society, recommend three antiretroviral drugs for both low- and high-risk exposures. There are no controlled data on the efficacy of any PEP regimen. There are also limited controlled data on the safety of ARVs in HIV-uninfected people, except for TDF + FTC from pre-exposure prophylaxis trials. It cannot be assumed that antiretroviral safety will be similar in HIV-infected and HIV-uninfected people, as illustrated by the severe toxicity of NVP when used in PEP. Therefore it is not possible to accurately determine risk-to-benefit ratios for PEP.

Life-threatening adverse drug reactions from currently recommended antiretroviral drugs are uncommon, probably occurring in about 1 in 1000 people, except for FTC and 3TC, which are considerably safer. People on the month-long course of PEP are at risk of life-threatening reactions, as many of them occur early (e.g. acute renal failure from TDF, severe hypersensitivity reactions). Therefore the number needed to harm (with life-threatening adverse drug reactions) may be similar to or lower than the number needed to treat to prevent one HIV infection when three-drug PEP is used following low-risk exposures.

In the absence of definitive data, clinical judgement needs to be exercised when balancing risks and benefits for PEP. It is reasonable to start three-drug PEP following an HIV exposure event. However, clinicians should have a low threshold to switch or stop offending antiretroviral drugs, should potentially severe adverse drug reactions occur. There may still be a place for two-drug PEP for very low-risk exposures.

## Prevention of exposure

Awareness of the risks and activities related to transmission of HIV, as well as availability of PEP and support, is critical, especially in an occupational setting. HCWs in traditional exposure environments often receive training regarding this hazard. Other potential areas where PEP should be available include, but are not restricted to, home-based carers, day centres and crèches, schools and prisons, where PEP exposure and treatment training are often poorly available.

Exposure to HIV occurs in a variety of situations, which HCWs should be aware of (see Box 1).

**BOX 1:** Potential HIV exposure situations.

**Exposure to HIV can occur in a vast variety of situations. Exposures where clinicians have requested advice regarding PEP, often where the source HIV and hepatitis status is unknown, include:**
Human bites or exposure to bloody phlegm during fights. Exposure at schools, including biting in crèche. Contact sports with blood exposure, such as rugby and boxing. Sharing needles during recreational drug use. Assaults with several people being stabbed with the same knife. Bullets travelling through one person and lodging in another. Animal attacks with repeated blood exposures on several people at once. Roadside and emergency services exposure – often not only by ambulance staff, but also police, bystanders who help. Exposure during home deliveries or during home-based care. Consensual sexual exposure, burst condoms, mucosal exposure during non-penetrative sex. Families, home-based carers. Catering, preparation and serving of food with blood contamination. Sitting on a needle in a movie theatre. ‘Venoterrorism’ – public attacks with needles. Unconscious drug user found in a room.
**The following exposures do not require PEP:**
Exposed individual is already HIV-positive. Source is confirmed HIV-negative by laboratory ELISA test and the window period has been excluded. Exposure to bodily fluids that do not pose significant risk of HIV transmission: tears, non-bloodstained saliva, sweat and urine.

HIV, human immunodeficiency virus; PEP, post exposure prophylaxis; ELISA, enzyme-linked immunosorbent assay.

### Prevention of HIV exposure in the workplace

Prevention of exposure to HIV and other blood-borne viruses in the workplace is the responsibility of both employer and employee. It is a legal requirement in many southern African countries for employers to provide a safe working environment and to ensure that employees adhere to workplace guidelines for infection control. South Africa has an extensive legal framework and comprehensive codes and guidelines dealing with this issue. Employers have specific and numerous responsibilities with regard to workplace safety and staff support. The meticulous recording and reporting of incidents is critical; this responsibility usually rests with a medical practitioner.

A broad range of professionals practising within the healthcare service and outside the Department of Health is at occupational risk of blood-borne viral exposure (see Box 2).

**BOX 2:** Who is at risk of occupational exposure to blood-borne viruses?

**Healthcare workers**
Doctors Dentists Nurses Traditional healers Phlebotomists Laboratory workers Physiotherapists Occupational therapists Paramedics
**Non-healthcare workers**
Firemen Commercial sex workers Teachers Prison warders Bar bouncers

## Special situations: Healthcare situations

**FIGURE 2:**
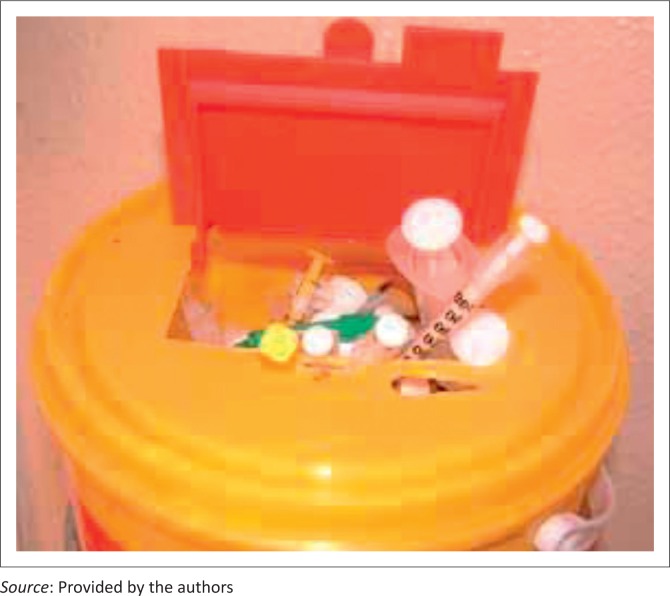
Unsafe sharps bin.

Occupational exposure involves potentially hazardous exposure to blood-borne viruses in the workplace:

- All occupational exposure should be regarded as preventable and hence deserving of investigation until proven otherwise.
- Standard precautions should be practiced in every setting where blood or infectious body fluid contact is possible. Gloves should be worn and, where appropriate, protective eyewear.
- Clean water or saline should be available to immediately irrigate any mucosal exposure or percutaneous injury. Use non-caustic soap. Only use water or saline if the exposure involves the eye.
- Needles should *not* be re-sheathed, and manipulation of the needle following withdrawal from the patient must be kept to the absolute minimum.
- Wherever possible, safety equipment for blood taking should be available, particularly in the hospital and clinic setting where the risk of exposure to HIV-infected blood is highest. It is imperative that the cost of cheaper equipment and disposal must be weighed against the potential increased risk of exposure that using such equipment entails.
- Needles and tools for any surgical practice, including traditional circumcision, should never be re-used without rigorous chemical disinfection/sterilisation according to national or local guidelines.
- All needles and sharp objects should be disposed of into a dedicated biohazard sharps bin. Syringes and other blunt instruments should *not* be disposed of in these bins, but rather in regulation biohazard bins for disposal of blunt biohazard objects.
- The number of sharps bins allocated to each workplace area will depend on the setting and the resources available. It is recommended that, in hospital settings, designated areas of high throughput of patients who require a large number of invasive procedures, such as intensive care and emergency departments, should have a ratio of sharps bins to beds of either 1:1 or 1:2. Isolation rooms should have their own sharps bins, as should any clinic area in which blood taking or invasive procedures are undertaken. The ratio of sharps bins to beds in open wards should ideally be 1:2, but at least one bin per bay.
- Once three-quarters full, the sharps bin should be sealed and disposed of to prevent obstruction of the opening; overfull bins are a risk factor for injury during subsequent sharps disposal (Figure 2). In resource-poor settings where sharps bins are unavailable, the safest and most practical method of sharps disposal should be practiced as per local or national guidelines.
- Within the hospital or clinic environment, it is the ultimate responsibility of that institution's infection control team to monitor and ensure that sharps bins are sealed when three-quarters full and disposed of correctly. However, on a day-to-day basis, this responsibility falls to the nursing sister in charge of the ward or clinic.
- Outside the healthcare setting, employers must take responsibility for such monitoring and enforce standard practice as laid out above.
- Best practice should be enforced with the aid of unions within the framework of occupational law to ensure that employers and employees create a safe working environment regarding prevention of blood-borne disease acquisition.

## Other situations

Post-sexual exposure prophylaxis is indicated for those who present within 72 hours of unprotected risky sexual activity including, but not limited to, penetrative intercourse and including, but not limited to, survivors of sexual assault. As a public health intervention, equal access to treatment of all sexual exposures, including rape, is essential to equality of prophylaxis and minimisation of HIV transmission:

- There is often considerable variation in clinical presentation of exposure situations, making it almost impossible to establish standard operating procedures for control of exposure, as may be possible in healthcare settings.
- The complications of criminal, civil and medico-legal elements, particularly in the case of criminally defined rape, are specialised elements of care that are beyond the scope of the present guideline. Applicable local guidelines should be consulted in such cases.
- PEP should be given as part of a package of care to women subjected to sexual assault, including support, emergency contraception and prophylaxis for additional sexually transmitted infections (STIs) in combination with psychological interventions (the details of this package of care are beyond the scope of this guideline – please consult applicable local guidelines).
- Other people who have been sexually assaulted need to have psychosocial issues addressed in combination with PEP as part of a package of care; this would include men, children and adolescents who have been sexually assaulted.
- Given the emotional and psychological trauma experienced by many of the patients who present after sexual assault, HIV-specific counselling may be appropriately delayed for 24–48 hours after onset of PEP regimens.
- It is recognised that the post-sexual assault situation has a high rate of therapy default, complicating all aspects of management.
- The choice of ARVs when several other agents are being utilised for pregnancy prophylaxis, STI syndromic management, and various medications to treat side-effects of trauma, is complicated. Despite the strong empirical arguments for triple ARV prophylaxis in this setting, a default to dual therapy with minimal short-term side-effects may be considered in individuals where there are concerns regarding adherence, or where an integrase inhibitor is not available as a third drug, with full disclosure of the potential risk of this strategy to the patient. Alternatively, more ARVs with better tolerability profiles are available and consideration should be given to switching the offending agent to a different one. There is no real evidence that a third drug in PEP gives any additional protection. In addition, prophylactic management, such as anti-emetics and antidiarrhoeals, should be considered as upfront therapy, given the high rate of PEP defaulters.

### Sexual exposure outside of a relationship, where disclosure concerning the exposure is not desired

This is a common and thorny problem faced by clinicians, with ethical and social implications. Marriage and long-term relationships are almost always assumed within our society to be monogamous, although ‘straying’ from the relationship is very common in all communities. Whilst a single episode of unsafe sex overall carries a low risk of HIV exposure, they may, should the exposed partner become positive, have a very high viral load during the seroconversion phase, and unprotected sex will carry a very high risk to the regular partner, whether PEP is given or not. We advise providing PEP for people who have had unprotected sex with a partner of known HIV-positive or unknown HIV status. Sudden cessation of regular sexual relationships or introduction of condoms can cause relationship disruption, and the exposed partner may be reluctant to do this. This situation raises issues concerning the duty of the HCW to disclose to the partner, and requires a very careful and individual approach. Any decision to disclose against the wishes of the exposed person to the partner must be carefully discussed with colleagues, representative organisations and medical defence organisations. Patients may require help with strategies around disclosure. Where the exposed individual is not putting their sexual partners at risk, for example by consistently using condoms, disclosure is not strictly necessary. Where the exposed individual is placing others at risk, the issue of disclosure or facilitated disclosure becomes relevant.

### Children

Principles around exposure for children are biologically similar to those for adults. As in pregnancy, newer agents have often not been tested in children and dosages might not have been determined. Therefore, recommended medications and dosages may differ and it is important to check doses carefully. Psychological and legal consent issues may differ from adults, and clinicians should be guided by local legislation. Children often do not give accurate histories, and anxious parents, especially in the context of possible sexual assault, may require significant counselling and careful referral.

Pre-mastication of food is commonly practised in both developed and developing countries, and several cases of transmission from caregivers to children have been described in the USA. This practice should be actively discouraged.

Another source of potential infection, through breast milk, is using wet nurses, as well as milk kitchens (the practice of pooling breast milk, and then transferring to bottles in healthcare facilities).

Finally, children are exposed to other children's behaviours, which may theoretically have transmission risks, such as biting. Most cases of biting do not pose a risk of HIV transmission in children, but if there is blood in the mouth of the biting individual (e.g. bleeding gums owing to gingivitis), or if the skin of the bitten child is breached, there is a theoretical risk of transmission.

Principles of PEP in children are the same as in adults, although managing parent anxiety is often a huge challenge.

## Selecting patients for antiretroviral interventions

### Potentially infectious material

The following should be regarded as infectious material:

- blood (and *any* bloodstained fluid, tissue or material)
- sexual fluids
- vaginal secretions
- penile pre-ejaculate and semen
- tissue fluids
- any fluid drained from a body cavity, including ascites; cerebrospinal, amniotic, rectal, peritoneal, synovial, pleural or pericardial fluids; and wound secretions
- breast milk.

Parenteral or mucosal exposure requires antiretroviral PEP intervention, as described in the present guideline.

In the absence of super-contamination with the above fluids, the following may be considered non-infectious:

- sweat
- tears
- saliva and sputum
- urine
- stool.

Exposure to HIV occurs in a vast variety of situations, which healthcare professionals (HCP) should be aware of (see Box 2). Exposure to non-infectious material requires reassurance but no PEP. A special circumstance involves human bites and punching. Where a bite or a punch has resulted in opening of the skin, PEP should be advocated, bearing in mind that in the case of human bites, the possibility that both the person bitten and the person who inflicted the bite were exposed to blood-borne pathogens.

PEP should be offered and initiated as early as possible after exposure, ideally within 72 hours, to all individuals with exposure that poses a risk for HIV transmission, and should be continued for one month. In exceptional cases involving high-risk exposures, PEP may be considered up to 7 days after exposure. It is advisable to discuss such cases with an experienced HIV clinician.

### Selecting antiretroviral regimens for post-exposure prophylaxis

#### Recommended post-exposure prophylaxis antiretroviral regimen

The choice of PEP combinations is based on available evidence in both prevention (including PrEP and PMTCT) and treatment settings; side-effect profiles; ease of use; local guidelines; and availability. In addition, the present PEP guidelines are aligned with the latest WHO PEP guidelines, released in December 2014, which now recommend three drugs as the preferred option for PEP, and no differentiation in regimen according to the type of exposure, namely occupational versus non-occupational. This approach is part of a move towards simplification of prescribing to improve availability of PEP and to reduce the time to PEP initiation. With the availability of less toxic and better tolerated drugs, providing a three-drug regimen supports simplified prescribing by removing the need to evaluate the risk of resistance, which was the basis upon which the decision to initiate two- versus three-drug PEP was previously made. While PEP completion rates are generally less than optimal, there is evidence that completion rates are similar when comparing two-drug to three-drug PEP (see Box 3).

**BOX 3:** Post-exposure prophylaxis recommendations.

**In adults and adolescents ≥35 kg:**
The preferred backbone for PEP is TDF + FTC/3TC.† Raltegravir (RAL) is the preferred third drug (except in pregnant women, where ATV/r is the preferred third drug). Alternative third drugs include ATV/r, LPV/r, DRV/r or EFV. It is imperative that the first dose of PEP is administered as soon as possible after exposure; if the 3 recommended drugs are not immediately available, use whatever suitable ARV medication is available to start. All PEP regimens must be administered for one month.

PEP, post exposure prophylaxis; TDF, tenofovir; FTC/3TC, emtricitabine/lamivudine; RAL, raltegravir; ATV/r, atazanavir/ritonavir; LPV/r, lopinavir/ritonavir; DRV/r, darunavir/ritonavir; EFV, efavirenz; ARV, antiretroviral.

†, AZT is poorly tolerated in PEP settings, whilst TDF + FTC/3TC has a better safety profile, and is similar in cost to AZT + 3TC. TDF + FTC/3TC is also recommended for PrEP. Owing to poor tolerability of AZT in PEP, d4T is well tolerated in short-term use and should be used where TDF is contraindicated.

#### Justification for three versus two drugs and for choice of antiretrovirals preferred for post-exposure prophylaxis

As in the previous guideline, the present update recommends that, where PEP is to be provided, three drugs should be administered. The basis of this recommendation is manifold.

Current North American Centers for Disease Control and Prevention (CDC) and UK guidelines are based on risk assessments in low-prevalence settings, with presumed exclusive clade B data. In contrast, the southern African situation is one of extremely high HIV prevalence (clade C), high volumes of patients, and an attendant very high number of exposures. The individual and cumulative risk of HIV transmission in this setting has never been quantified. There are limited data suggesting that clade C is more infectious in the sexual exposure setting. We assume that this risk is significantly higher than in other settings, and the exposed individual should therefore be treated appropriately. For key populations, such as men who have sex with men (MSM), intravenous drug users, prison populations and others, the risk is even higher than penile-vaginal penetration, which adds further weight to this recommendation.

Whilst previous guidelines advocated two or three drugs based on clinician assessment of risk, the SA HIV Clinicians Society has recommended three drugs in all exposure situations since 2008. There is no evidence backing the use of two drugs over the single-agent AZT. We further note that the PMTCT trials suggest no added advantage of adding 3TC to AZT, a finding replicated in various cohort PMTCT studies. Several PrEP studies have shown TDF + FTC to be superior to TDF alone in preventing HIV infection occurring in study participants. In addition, the use of triple-therapy ART regimens has been shown to have significant benefit in comparison with dual therapy in treatment settings. Whilst no evidence exists to support the use of such combinations in humans in PEP scenarios, all current PEP guidelines advocate triple prophylaxis regimens in ‘high-risk scenarios’. The argument is therefore not one of two or three drugs, but of what constitutes ‘high-risk scenarios’. Southern Africa, with its high prevalence, large numbers of patients and high number of exposures, should be considered a high-risk scenario.

Of particular contention are mucous membrane exposures and oral sex scenarios, which are associated with lesser risk. The CDC guideline is based on a single known transmission out of almost 10 000 reported incidents. Again, no evidence of risk is available in our setting, but evidence of significantly higher exposures in comparison to the US setting (blood spatters on eyeglasses, masks in low-, medium- and high-risk procedures) is available. Furthermore, blood risk exposures are chronically underreported, a factor that is likely to be particularly true of injuries that are deemed to carry a lesser risk, meaning the incidence may be greater than we think. For these reasons, coupled with the known high background HIV prevalence, we advocate three-drug PEP in these scenarios. However, the extremely low risk of transmission via these routes should be discussed with the exposed individual. On the other hand, the advocated PEP triple regimen is very well tolerated compared with previously used regimens, and the exposed individual may opt to take the PEP regimen despite the low risk of transmission via mucous membrane exposure.

Finally, the risk of side-effects increases when additional agents are added to PEP regimens. Three-drug regimens carry more risk of side-effects than two-drug regimens. With the availability of more tolerable drugs, potential side-effects are fewer, and can be anticipated and managed proactively, to ensure the full month of PEP is completed. Integrase inhibitors have minimal side-effects, and are a well tolerated third drug option.

#### Justification for choice of antiretrovirals preferred for preferred for post-exposure

AZT-containing regimens carry such a significant side-effect profile that this agent should be avoided, and with the reduction in cost of TDF combinations, the recommended NRTI backbone is now TDF with FTC/3TC, preferably in a FDC. There is no evidence that prevention of HIV transmission by AZT in the setting of PEP is effected by anything other than its inhibition of viral replication. This supports the use of TDF, whose potency of action is equivalent to AZT, yet which is far better tolerated over one month of therapy, as a recommended NRTI in PEP. Whilst the risk of adverse events is undeniably real, it must be balanced against the often unquantifiable but equally real risk of transmission associated with high HIV prevalence, high individual viral load levels, and high levels of exposures in occupational and non-occupational settings.

TDF is usually avoided in patients on ART with renal failure or eGFR < 50 mL/min. In the setting of PEP, the duration of TDF administration is short. Comparative data from three randomised trials for ART and PrEP and from observational studies with PEP support the use of TDF + FTC/3TC as the preferred backbone in PEP. Indirect comparisons between AZT + 3TC versus TDF + FTC/3TC across 15 studies demonstrate less PEP discontinuation due to adverse events in individuals receiving TDF + FTC/3TC than AZT + 3TC. However, where there are concerns regarding the use of TDF, d4T is very well tolerated when it is used for short periods and, given the poor tolerability of AZT in PEP regimens, would be the recommended NRTI to use in such cases.

There may be a risk of hepatic flares in individuals chronically infected with HBV who discontinue PEP containing TDF, 3TC or FTC, as has been seen in some patients on ART who switch away from these drugs. Such individuals should be monitored for hepatic flare if these drugs are not continued for HBV treatment. Where HBV testing is available, those with unknown HBV status should be tested for active HBV infection, to assess the need for ongoing HBV therapy.

In terms of third drug options, there are many agents which may be suitable for use in PEP but which may have limitations such as cost and availability in low- and middle-income countries, such as in the southern African region. There are studies that provide data on lopinavir + ritonavir (LPV/r), ATV/r, darunavir + ritonavir (DRV/r) and raltegravir (RAL) as part of triple-combination PEP, but offer little guidance on their efficacy.

The present guideline recommends using RAL as the third drug, with ATV/r, LPV/r, DRV/r or efavirenz (EFV) as alternatives where RAL is not available or cannot be used. RAL in combination with TDF + FTC/3TC (as an FDC) is the preferred PEP regimen on account of its tolerability, potency, convenience and minimal drug interactions. This regimen differs from WHO recommendations, which advocate the use of LPV/r or ATV/r as the third drug in PEP, as they are currently used in ART and are widely available in low- and middle-income countries, which is not always the case with RAL or DRV/r because of the higher cost of these agents.

If the three recommended drugs are not immediately available, this should not delay the initiation of PEP. It is imperative that PEP is started as soon as possible after exposure; in cases where the recommended 3 drugs are not immediately available, an alternative 3-drug combination can be given immediately. However, the patient must not leave without a full month's supply of the recommended 3 drugs.

There are other newer drugs that might be useful as part of a PEP regimen, but there are no data supporting their use in PEP specifically. The drugs include dolutegravir (high potency, tolerability and once-daily dosing); rilpivirine (high tolerability and low cost) and elvitegravir (tolerability and convenient coformulation). Once dolutegravir becomes available in South Africa, it is likely to replace RAL as the third drug of choice in PEP, with advantages being once-daily dosing (compared with RAL which is twice a day) and the possibility of dolutegravir-containing FDCs.

EFV is currently the preferred third drug in first-line ART, and is generally well tolerated in the long term; however, it is associated with early nervous system and psychiatric side-effects that limit its use in PEP. Owing to the possibility of high levels of anxiety in the exposed individual, EFV should only be used as the third drug where other drugs cannot be used.

Nevirapine (NVP) and abacavir (ABC) are not recommended for PEP, on account of their risk of serious side-effects.

#### Justification for duration preferred for post-exposure

A one-month prescription for ARVs should be provided for PEP. This is supported by animal study data that demonstrate that a full 28-day course is necessary to achieve maximum benefit from the intervention and prevent seroconversion.

Before the widespread availability of rapid HIV tests, starter packs of PEP were dispensed to ensure testing and counselling could be completed, accommodating the longer turnaround time of the available tests. However, it is now recommended that the full one-month course is dispensed to improve completion rates, which are lower amongst those exposed who received partial prescriptions than those receiving the full course at the initial visit.

Providing the full course removes the need for a 3-day follow-up visit, reducing the burden on facilities, as well as being more convenient for exposed individuals. However, they should be fully informed about the side-effects of the PEP regimen, and advised to return to the facility if they have any concerns, side-effects or adherence problems prior to their scheduled follow-up visit. A follow-up appointment for 2 weeks should be scheduled, and at this visit any side-effects should be proactively identified and managed, and appropriate counselling provided. The next appointment should be scheduled for 6 weeks post-exposure, where appropriate laboratory tests will be done (as per Table 4).

**TABLE 2:** Selecting patients for preferred for post-exposure interventions.

**Type of exposure**	**Status of the source**		
	**HIV-positive**	**Unknown**	**HIV-negative**
Percutaneous exposure to blood or potentially infectious fluids	Triple prophylaxis	Triple prophylaxis	No PEP
Mucous membrane exposure, including sexual exposure, mucocutaneous splash or open wound contact, with blood or potentially infectious fluids	Triple prophylaxis	Triple prophylaxis	No PEP
Mucous membrane exposure, including sexual exposure, mucocutaneous splash or open wound contact, with non-infectious fluids	No PEP	No PEP	No PEP

PEP, post exposure prophylaxis

**TABLE 3:** Doses of antiretrovirals for HIV preferred for post-exposure in adults and adolescents.

**Generic name**	**Dose**
Tenofovir (TDF)	300 mg once daily
Lamivudine (3TC)	150 mg twice daily or 300 mg once daily
Emtricitabine (FTC)	200 mg once daily
Stavudine (d4T)	30 mg twice daily
Raltegravir (RAL)	400 mg twice daily
Atazanavir/ritonavir (ATV/r)	300/100 mg once daily
Lopinavir/ritonavir (LPV/r)	400/100 mg twice daily or 800/200 mg once daily†
Darunavir + ritonavir (DRV/r)	800/100 mg once daily or 600/100 mg twice daily
Efavirenz (EFV)	600 mg at night (400 mg if weight < 40 kg)

*Source:* Adapted from World Health Organization. Guidelines on post-exposure prophylaxis for HIV and the use of co-trimoxazole prophylaxis for HIV-related infections among adults, adolescents and children: Recommendations for a public health approach: December 2014 supplement to the 2013 consolidated ARV guidelines. Geneva: World Health Organization; 2014

†, Once-daily dosing can be considered as an alternative for adults, but more data needed for children and adolescents.

**TABLE 4:** Timing of bloods pre- and post-preferred for post-exposure.

**Laboratory tests**	**Source: Baseline**	**Exposed**			
		**Baseline**	**2 weeks**	**6 weeks**	**3 months**
HIV	Rapid test *plus* 4th-generation ELISA	Rapid test *plus* 4th-generation ELISA	-	4th-generation ELISA	4th-generation ELISA
HBV	HBsAg	HBsAb‡	-	-	HBsAg‡
HCV	HCV Ab†	HCV Ab§	-	HCV PCR§	-
Syphilis	RPR/TP Ab	RPR/TP Ab§	-	-	RPR/TP Ab§
Creatinine	-	If TDF part of PEP	If TDF part of PEP	-	-
FBC	-	If AZT part of PEP	If AZT part of PEP	-	-

HBV, hepatitis B virus; HCV, Hepatitis C virus; FBC, full blood count; ELISA, enzyme-linked immunosorbent assay; HBsAg, hepatitis B surface antigen; Ab, antibody; RPR, rapid plasma reagin; TP, *Treponema pallidum*; HBsAb, hepatitis B surface antibody; TDF, tenofovir; PEP, post exposure prophylaxis; AZT, zidovudine; PCR, polymerase chain reaction.

†, Only if high risk for HCV or source unknown; ‡, can be omitted if exposed individual known to be protected (natural immunity or vaccination); §, only if source patient was positive.

## Routine baseline and follow-up investigations

### Investigating the source individual

Where the source individual is known, every effort must be made to gain their voluntary, informed consent to have the necessary laboratory tests performed, in accordance with Health Professions Council of South Africa (HPCSA) guidelines and national policy regarding HIV testing. If the source individual is unknown, unavailable for testing, or refuses testing after appropriate counselling, the default position should be that the source is seropositive for HIV. If a source individual is unable to give consent because of an impaired level of consciousness, national guidelines allowing testing in such circumstances should be followed. Testing of the source should be undertaken as soon after the injury as possible. Testing of needles, sharps or other samples that have been implicated in the exposure is not recommended, even when the source is unknown or refuses testing. Such investigations are unreliable and pose a risk of further exposure to the HCWs undertaking the testing.

The tests that should be performed on blood from the source individual are shown in Table 4. If the source is found to be positive on any of the tests undertaken, they should receive post-test counselling and either be treated or referred to their local healthcare facility for further management.

#### HIV testing

A nationally approved HIV test should be performed by a HCW who is trained in this procedure, with pre- and post-counselling, and formally documented.

A positive rapid test should be confirmed, as per national guidelines, and the source patient managed as per guidelines. If the diagnosis of HIV is confirmed in the source patient, they must be linked with treatment and care services immediately.

For source patients on antiretrovirals, HIV RNA PCR should be performed where available. If the viral load is not fully suppressed, genotypic testing should be considered, although this is of uncertain value. This test should, however, not delay initiation of PEP. Detectable viral load results should be discussed with an expert. If viral load testing and/or genotyping are not available, and if resistance is suspected, a boosted PI should always be used as the third drug.

As the plasma viral load measures only the level of cell-free virus in peripheral blood and so an undetectable viral load does not exclude low-level viraemia, the possibility of transmission from a source patient with an undetectable viral load is not eliminated. In such cases, the exposed individual should still be offered PEP and appropriate follow-up.

#### HIV and hepatitis B virus testing

Testing of the source for hepatitis B surface antigen (HBsAg) can be omitted when the exposed individual is known to be protected from hepatitis B acquisition by natural immunity or vaccination.

#### Hepatitis C virus testing

Hepatitis C virus (HCV) is rare in SA and we do *not* recommend testing unless the source individual is an intravenous drug user, MSM, haemophiliac or from a high HCV prevalence setting, or where the source is unknown. In such cases, the source should be tested for HCV Ab. If the source is HCV-negative, the exposed individual should be tested at baseline to assess their own HCV status, and no further HCV testing will be necessary in further follow-up. However, where the source is HCV-positive and the exposed individual is HCV-negative at baseline, HCV PCR testing should be done at 6 weeks.

#### Other blood-borne pathogens

Syphilis: routine testing of source should be performed. Malaria: malaria blood films should *not* be routinely sent from source patients, unless there is clinical suspicion that the source has malaria.

### Investigating the exposed individual

It is strongly recommended that any investigation on the blood of an exposed person should be requested and taken by an independent third party. If infection is proven, baseline investigation for blood-borne viruses forms a vital part of any future compensation claim.

#### HIV testing

Pre- and post-test counselling should be offered to all exposed persons at all testing facilities. A baseline HIV rapid test, followed by 4th-generation ELISA as confirmation should be performed and the results carefully documented. As many cases have medico-legal or occupational claims implications, it is recommended that formal laboratory testing be done in all cases. Confirmatory testing of a positive result should be undertaken per standard guidelines.

Follow-up testing for HIV seroconversion should be undertaken at 6 weeks and 3 months post-exposure. We do not advocate routine testing of an exposed worker at 6 or 12 months, as current ELISA tests (4th generation) have reduced the window period considerably.

In exposed individuals, testing beyond 3 months is advised in the following settings:

- ongoing high-risk behaviour
- a specific exposure incident within the last few months can be identified
- HIV status at 3 months is indeterminate.

Viral load or p24 antigen testing is not recommended in the setting of PEP. Quantitative viral loads may yield false-positive results, and may cause substantial anxiety. Seroconversion on PEP is extremely rare; any exposed individual thought to be experiencing a seroconversion illness on PEP should be discussed with an HIV specialist physician for advice. If an exposed individual tests HIV-positive at any stage, they should be linked to treatment and care services as soon as possible.

#### Hepatitis B testing

The risk of transmitting the HBV is higher than that of HIV in most exposures, especially in the healthcare environment. If the exposed worker has had prior HBV infection or has been vaccinated and is a known responder, then no investigation or post-exposure therapeutic intervention for HBV is required.

If the source individual tests HBsAg-negative and the exposed individual is not vaccinated or does not know their vaccination/antibody status, they should be referred to a local facility for testing and vaccination. In the case of exposure to an HBsAg-positive source, the options for management of unvaccinated individuals or those whose status is unknown are as detailed in Table 5.

**TABLE 5:** Management of an individual exposed to an HBsAg-positive or unknown source.

**Vaccinated status of exposed**	**HBV vaccine**	**HBIG (0.06 mL/kg)**	**HBsAb**
Previous vaccination; known responder	None	None	Not done
Not vaccinated	1st dose stat and proceed to accelerated schedule	If HBsAb < 10 IU/mL, give stat HBIG and repeat at 1 month	If HBsAb > 10 IU/mL, no treatment
	(0, 1 and 6 months)		
Incomplete vaccination or unsure	Complete depending on documentation, or restart	Single dose stat	-
	0, 1 and 6 months		
Vaccinated; unknown response	Single booster stat		
Non-responder to prior vaccination	1st dose stat and proceed to accelerated schedule	1 dose stat, repeat after 1 month	HBsAb < 10 IU/mL
	0, 1 and 6 months		
Previously vaccinated with four doses or two completed vaccine series; non-responder	Consider alternative vaccine	-	-

Comment: HBIG and HBV vaccine can be administered concomitantly at different sites.

HBV, hepatitis B virus; HIV, human immunodeficiency virus; HBIG, hepatitis B immunoglobulin; HBsAb, hepatitis B surface antibody.

#### Hepatitis C virus testing

- Only if the source individual is an intravenous drug user, MSM, haemophiliac or from a high HCV prevalence setting, or where the source is unknown.
- In such cases, the source should be tested for HCV Ab. If the source is HCV-negative, the exposed individual should be tested at baseline to assess their own HCV status, and no further HCV testing will be necessary in further follow-up.
- However, where the source is HCV-positive and the exposed individual is HCV-negative at baseline, HCV PCR testing should be performed at 6 weeks.

#### Other blood-borne pathogens

Malaria: routine testing of an individual who has been exposed to a source is *not* recommended unless the source is symptomatic.

#### Sexually transmitted infections

In cases of sexual exposure, exposure to other sexually transmitted infections might have occurred. If symptomatic, manage syndromically. Otherwise, appropriate prophylaxis should be provided to the exposed individual. However, these guidelines do *not* deal with the comprehensive management of sexual assault. Appropriate guidelines should be consulted for sexual assault cases.

#### Pregnancy

All exposed women should be screened for pregnancy at the time of the incident and subsequent follow-up. Emergency contraception should be offered to all women of childbearing age who present after accidental exposure or sexual assault, in line with relevant guidelines.

#### Tetanus

Individuals who have wounds such as abrasions, cuts or bites should be asked about their tetanus immunisation status, and be offered immunisation if appropriate.

### Follow-up: Monitoring for adverse drug reactions

#### Side-effects

The present guideline's emphasis on appropriate choice of agents to minimise side-effects, on close management of the individual patient through the PEP process, and on the aggressive prophylactic and therapeutic management of side-effects, allows a great deal of amelioration of the side-effect risk. This approach then tips the risk/benefit balance back towards the use of the most virologically potent regimens available, namely three-drug regimens. Management guidelines to minimise exposure risk also form a large part of the present guideline, but once exposure has occurred, management of side-effects is almost always achievable, whilst the attendant risks are not. For common side-effects with the preferred and alternative PEP antiretroviral agents, see Table 6. Mainly shorter-term side-effects such as nausea, vomiting and headaches which are transient or can be managed have been included in the table. Longer-term toxicities that are unlikely to be seen with one-month PEP regimens are not included (e.g. lipoatrophy, hyperlactataemia, steatohepatitis).

**TABLE 6:** Common or severe adverse drug reactions of antiretrovirals that may be used for preferred for post-exposure.

**Generic name**	**Drug class**	**Common or severe adverse drug reactions**
Tenofovir (TDF)†	NtRTI†	Well tolerated. Nephrotoxicity: avoid in individuals with pre-existing renal disease†
Lamivudine (3TC)†	NRTI†	Well tolerated†
Emtricitabine (FTC)†	NRTI†	Well tolerated†
Raltegravir (RAL)†	InSTI†	Well tolerated. Occasional skin hypersensitivity, rhabdomyolysis (rare)†
Stavudine (d4T)	NRTI	Well tolerated
Zidovudine (AZT)	NRTI	Nausea, vomiting, headache, insomnia and fatigue common, anaemia, neutropenia
Efavirenz (EFV)	NNRTI	Central nervous system symptoms (vivid dreams, problems with concentration, dizziness, confusion, mood disturbance, psychosis, insomnia, somnolence), rash, hepatitis
Rilpivirine (RPV)	NNRTI	Well tolerated. Rash, hepatitis, central nervous system symptoms (all uncommon)‡
Atazanavir (ATV)	PI	Unconjugated hyperbilirubinaemia (visible jaundice in some patients), rash, hepatitis (uncommon)§
Lopinavir/ritonavir (LPV/r)	PI/r	Gastrointestinal intolerance, nausea, vomiting and diarrhoea are common§
Darunavir (DRV)	PI	Diarrhoea, nausea, headache. Rash (contains sulphonamide moiety: use with caution in patients with sulpha allergy)§

NtRTI, nucleotide reverse transcriptase inhibitor; NRTI, nucleoside reverse transcriptase inhibitor; InSTI, integrase strand transfer inhibitor; NNRTI, non-nucleoside reverse transcriptase inhibitor; PI, protease inhibitor; PI/r, ritonavir-boosted protease inhibitor

†, Preferred antiretrovirals for post exposure prophylaxis; ‡, drug interactions need to be considered; §, must be boosted with ritonavir; drug interactions.

#### Comorbidities

Patients with significant comorbidities should have regular monitoring of any relevant investigations during therapy. No additional investigations are warranted in otherwise healthy individuals.

#### Medical comorbidities and antiretroviral selection for preferred for post-exposure

Although many of the comorbid conditions listed in Table 7 do not preclude the use of certain ARVs, increased monitoring of the comorbid condition may be necessary during the one-month course of PEP. Moreover, whenever a safer regimen is available with equal efficacy, that regimen should be used in preference.

**TABLE 7:** Comorbidities affecting choice of antiretrovirals for preferred for post-exposure.

**Comorbidity**	**Drug**	**Complication**
Tuberculosis	LPV/r	Double the dose of LPV/r if patient is on rifampicin
Epilepsy	PIs	PIs increase the level of a number of commonly used anticonvulsants
	EFV	Increased risk of seizures
Psychosis	EFV	Increased risk of psychiatric symptoms
Insomnia	PIs	St John's Wort reduces all PI levels
Migraine	Migraine	All PIs increase risk of ergotism with ergotamine coadministration
Renal failure	NRTIs	Avoid TDF if creatinine clearance < 60 mL/min. Dose adjust AZT, d4T and 3TC
Hypertension	PIs	PIs increase levels of calcium channel blockers. RTV increases beta blocker levels
Asthma	PIs	PIs decrease theophylline levels
DVT/PE	PIs	Increase warfarin levels, leading to risk of bleeding

LPV/r, lopinavir/ritonavir; PI, protease inhibitor; EFV, efavirenz; NRTI, nucleoside reverse transcriptase inhibitor; TDF, tenofovir; AZT, zidovudine; d4T, stavudine; 3TC, lamivudine; RTV, ritonavir; DVT/PE, deep vein thrombosis/pulmonary embolus.

#### Drug safety in pregnancy

In pregnancy, the benefits of ARVs must be weighed against the risks of adverse events to the woman, foetus and newborn. Data regarding the use of most ARVs during pregnancy are limited, and usually not of high quality. Much of the information regarding the use of ARVs in pregnancy is from the Antiretroviral Pregnancy Registry that, by virtue of the voluntary nature of registration, introduces a selection bias.

As there is less information regarding the use of RAL in pregnancy than ATV/r, the present guideline recommends that ATV/r be the third drug of choice for PEP during pregnancy. Both ATV/r and RAL are Food and Drug Administration (FDA) pregnancy category C. Table 8 provides information on the use of the drugs recommended for PEP during pregnancy.

**TABLE 8:** Drug safety in pregnancy.

**Drug**	**Comment**
Tenofovir (TDF)	High placental transfer. No evidence of human teratogenicity. All have anti-HBV activity, therefore risk of hepatitis flare if stopped.
Emtricitabine (FTC)	
Lamivudine (3TC)	
Stavudine (d4T)	High placental transfer. No evidence of human teratogenicity.
	Do not use with ddI (risk of lactic acidosis) or AZT (both thymidine analogues).
Zidovudine (AZT)	High placental transfer. No evidence of human teratogenicity.
	Do not use with d4T (both thymidine analogues).
Raltegravir (RAL)	High placental transfer. Insufficient data to assess human teratogenicity.
	Case report of markedly elevated liver transaminases in late pregnancy.
Dolutegravir (DTG)	Unknown placental transfer. Insufficient data to assess human teratogenicity.
	No data on use in pregnancy.
Atazanavir (ATV)	Low placental transfer. No evidence of human teratogenicity.
	Increased dosing in T2/3?
	Non-pathologic neonatal hyperbilirubinaemia.
Lopinavir (LPV)	Low placental transfer. No evidence of human teratogenicity.
	Once daily dosing not advised during pregnancy.
	Avoid oral solution owing to alcohol and propylene glycol content.
Darunavir (DRV)	Low placental transfer. Insufficient data to assess human teratogenicity.
	Less experience in pregnancy than LPV/r and ATV/r.
Ritonavir (RTV)	Low placental transfer. No evidence of human teratogenicity.
	Not used for antiretroviral effect, but in lower doses as PI booster in combination with other PIs.
	Avoid oral solution owing to alcohol content.
Efavirenz (EFV)	Moderate placental transfer.
	Potential foetal safety concerns. No increase in overall birth defects with T1 exposure in humans.

HBV, hepatitis B virus; ddI, didanosine; T2/3, trimester 2/3; PI, protease inhibitor.

### Key issues regarding counselling

#### Adherence

PEP studies report low completion rates, often less than 60% for all populations, but especially adolescents, and PEP following sexual assault. Adherence counselling has been shown to improve adherence in HIV-positive individuals starting ART. Three RCTs comparing standard care counselling to enhanced adherence packages in improving adherence to PEP were identified and reviewed. The enhanced package included individual baseline needs assessments, adherence counselling, and education sessions and telephone calls. The combined effect of the enhanced intervention improved adherence and completion rates compared with standard of care counselling. Based on this finding, it is likely that some of the methods used to improve ART adherence may well be effective in PEP, such as peer support, alarms, text messages and phone calls.

#### Anxiety management

Anxiety should not simply be dismissed as baseless with simple reassurance. HIV remains a ‘dread disease’, despite the success of ART, because it is sexually transmitted, still accounts for significant mortality and morbidity, and has extensive stigma associated with it.

Anxiety management must be part of the adherence or follow-up support, and may need several interventions. Simple telephonic contact and reassurance is almost always adequate.

The intervention must be individualised, but the following approaches should be integrated:

- Contextualise the risk: emphasise that acquisition of HIV is unusual through a single exposure, unless the injury is severe (sexual assault, blood transfusion of an infected unit, severe penetrating injury with infected tissue).
- Even in the case of severe exposure or injury, where PEP is used timeously and the course completed, the risk of transmission is extremely low.

#### Risk-taking interventions

Counselling should be non-judgemental, practical and solution-focussed. PEP is an ideal time to deal with risk-taking environments, whether unsafe sex (e.g. a one-night stand with unprotected sex), poor occupational health (e.g. overfull sharps bins) or other (e.g. injecting drug use). Addressing occupational risk must be practical (e.g. report overfull bins to infection control, do not tell an exhausted nurse to ‘be more careful’).

Secondary prevention to prevent harm to others (e.g. risk to a spouse after sex with a third party) must be addressed. Exposed individuals should be counselled on how to prevent transmission to others, until they undergo the three-month post-exposure test following PEP:

- use of condoms to protect sexual partners
- to prevent mother-to-child-transmission (MTCT), avoid pregnancy (provide emergency contraception if necessary) and avoid breastfeeding if possible (high risk of transmission via breast milk during the 3 months following seroconversion demonstrated in a study from Zimbabwe)
- safe injecting practices
- avoid blood and tissue donation.

Consider offering PrEP to exposed individuals where chronic exposure to HIV is unavoidable or likely to continue (e.g. sex workers). Current evidence indicates that PrEP is effective as part of combination prevention approaches, provided it is used correctly. For more information, consult the Southern African HIV Clinicians Society guidelines on the safe use of PrEP in MSM and the US Department of Health and Human Service DHHS clinical practice guideline.^11,12^
